# Adherence to antiretroviral therapy among women living with HIV/AIDS in the interior of the Brazilian state of Pará: cross-sectional study

**DOI:** 10.1590/1516-3180.2020.0370.R1.18112020

**Published:** 2021-04-05

**Authors:** Paula Gabrielle Gomes Candido, Bruna Melo Amador, Fabricio Ferreira Silva, Floriacy Stabnow Santos, Luiz Marcelo de Lima Pinheiro, Aldemir Branco de Oliveira

**Affiliations:** I Nurse and Master’s Student of Health and Technology, Universidade Federal do Maranhão (UFMA), Imperatriz (MA), Brazil.; II MSc. Manager of Health Programs, Municipal Health Department, Bragança (PA), Brazil.; III Undergraduate Student, Universidade Paulista (UNIP), Imperatriz (MA), Brazil.; IV PhD. Professor, Health Sciences Center, Universidade Federal do Maranhão (UFMA), Imperatriz (MA), Brazil.; V PhD. Professor, School of Biological Sciences, Campus do Marajó, Universidade Federal do Pará (UFPA), Soure (PA), Brazil.; VI MSc, PhD. Professor, Institute of Coastal Studies, Universidade Federal do Pará (UFPA), Bragança (PA), Brazil.

**Keywords:** HIV, Acquired immunodeficiency syndrome, Therapeutics, Anti-retroviral agents, Women’s health, Aids, Treatment, Antiretroviral drugs, Health service, Amazon region

## Abstract

**BACKGROUND:**

High prevalence of human immunodeficiency virus (HIV) infection and occurrence of drug-resistant strains have been recorded in northern Brazil. Abandonment of treatment and insufficient and inadequate adherence to antiretroviral therapy (ART) among people living with HIV/AIDS (PLWHA) have been recorded in the metropolitan area of Belém, the capital of the state of Pará.

**OBJECTIVES:**

To identify the sociodemographic profile and level of adherence to ART among women seen at a referral unit in the interior of Pará, northern Brazil.

**DESIGN AND SETTING:**

Cross-sectional study at a referral unit for care for PLWHA.

**METHODS:**

We included 86 women living with HIV/AIDS (WLWHA) in the Rio Caeté integrated region, northeastern Pará. Social, demographic and behavioral information, as well as the ART level, were obtained using forms that have been described in the scientific literature. Logistic regression models were used to assess associations of variables with ART.

**RESULTS:**

Most WLWHA were single (52.4%), young (47.7%) and heterosexual (97.7%), had low levels of education (63.0%), were unemployed (69.8%), had one sexual partner (75.7%), used condoms (46.7%) and were not using either licit drugs (68.7%) or illicit drugs (89.6%). Their adherence level was classified as insufficient , and only their viral load showed an association with ART.

**CONCLUSIONS:**

The participants’ low level of education and poor socioeconomic conditions may have been interfering with their adherence to ART. Such influences can be minimized through multiprofessional interventions that take the individuality of women served by the healthcare service into consideration.

## INTRODUCTION

In Brazil, the epidemic scenario of infection by the human immunodeficiency virus (HIV) and the acquired immunodeficiency syndrome (AIDS) has undergone several changes over time.^1^ Currently, involvement of socially more vulnerable populations, non-homogeneous distribution of the disease among Brazilian regions, especially with increased numbers of notifications in small and medium-sized municipalities, and the growing number of HIV-infected women are hallmarks of this epidemic.^2^^,^^3^^,^^4^ These characteristics indicate that the Brazilian healthcare system presents deficiencies with regard to prevention and treatment of HIV infection, especially in municipalities and population groups located in more distant and difficult-to-access areas, as occurs in northern Brazil.^1^^,^^2^^,^^5^^,^^6^^,^^7^

Over the last ten years, the northern region of Brazil has shown an upward trend in the rate of HIV/AIDS detection: 16.4 cases per 100,000 inhabitants were registered in 2007 and 23.6 cases per 100,000 inhabitants were registered in 2017 (an increase of 44.2%), with the state of Pará contributing an increase of 55%.^2^ In this Brazilian state, high prevalences of HIV infection and occurrences of drug-resistant strains have been recorded among people living with HIV/AIDS (PLWHA) in the cities of Belém and Bragança.^7^^,^^8^^,^^9^^,^^10^ Abandonment of treatment and insufficient and inadequate adherence to antiretroviral therapy among PLWHA has also been recorded in the city of Belém.^11^

In northern Brazil, there are few studies on adherence to antiretroviral therapy (ART) among PLWHA. Proper use of ART enables reductions in morbidity and mortality rates and significant improvements in quality of life and life expectancy among PLWHA.^12^^,^^13^^,^^14^ Multiple factors have been correlated with abandonment of treatment and with insufficient or inadequate adherence to ART, such as: complexity of therapeutic methods and their respective effects, socioeconomic status, low level of education, family beliefs and values, affective social support, PLWHA’s relationships with doctors and other professionals in healthcare services, use of psychotropic drugs and mental disorders.^1^^,^^12^^,^^14^^,^^15^ Non-adherence or low adherence to treatment and incorrect use of ART are considered to be strong threats to the effectiveness of treatment among PLWHA. These situations have been directly correlated with therapeutic failure. They facilitate proliferation of HIV strains that are resistant to existing drugs, which gives rise to a need for combined use of other drugs.^14^^.^^16^ Accurate assessment of adherence and other aspects of this process is essential for proper planning of care for PLWHA and for development of effective strategies for adherence to ART.

## OBJECTIVES

The objectives of the present study were to identify the sociodemographic profile of women attended at a referral unit for specialized HIV/AIDS care in the Rio Caeté integrated region, Pará, northern Brazil, and their level of adherence to ART.

## METHODS

### Study area

**Figure 1.**
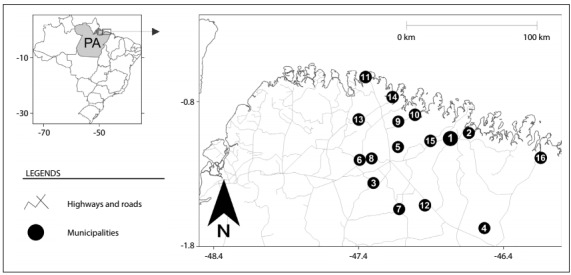
Geographical locations of the 16 municipalities in the Rio Caeté integrated region, Pará (PA), northern Brazil. Points = municipalities: Bragança (1), Augusto Corrêa (2), Bonito (3), Cachoeira do Piriá (4), Capanema (5), Nova Timboteua (6), Ourém (7), Peixe Boi (8), Primavera (9), Quatipuru (10), Salinópolis (11), Santa Luzia do Pará (12), Santarém Novo (13), São João de Pirabas (14), Tracuateua (15) and Viseu (16).

This study was conducted among women assisted at a specialized care service (SCS) in the city of Bragança. This SCS formed a reference unit for specialized care relating to HIV/AIDS in the Rio Caeté integrated region, which is located in the northeast of the state of Pará, northern Brazil.^17^ This region has the second highest demographic density in the state of Pará, with a population of around 495,000 inhabitants, distributed in 16 municipalities: Augusto Correa, Bonito, Bragança, Cachoeira do Piriá, Capanema, Nova Timboteua, Ourém, Peixe-Boi, Primavera, Quatipuru, Salinópolis, Santa Luzia do Pará, Santarém Novo, São João de Pirabas, Tracuateua and Viseu (Figure 1). Fishing, agriculture and extraction of natural resources, such as crabs, shrimps, wood and minerals, are the main economic activities developed in this region. Most of these municipalities have low human development indexes (HDI) and a variety of socioeconomic problems, such as high illiteracy rates, informal work and crack use, and more than half of the population is below the poverty line.^7^^,^^17^^,^^18^

### Study design and sampling

This cross-sectional study consisted of a convenience sample (non-probabilistic). Hence, participants living with HIV/AIDS were selected when they attended the SCS for medical consultations or medication withdrawal. Specifically, all women aged 18 years or over who had previously been diagnosed with HIV/AIDS, had been receiving ART for more than three months and were being attended at the SCS of the Rio Caeté integrated region were invited to participate in this study. Women who refused to complete the data-gathering questionnaires, pregnant women and women with cognitive impairment and/or with some debilitating infection that would make it impossible to answer the questionnaires were excluded.

Thus, between October and December 2018, 110 women living with HIV/AIDS (WLWHA) were attended at the SCS of the Rio Caeté integrated region, Pará. All of these women were invited to participate in this study. However, 24 women were not included: nine were just starting ART and 15 did not agree to answer the questions of the data-gathering instruments. Therefore, the sample was composed of 86 WLWHA, i.e. 78.2% of the WLWHA attended at the SCS of the Rio Caeté integrated region during the study period.

### Data-gathering

Data on the participants were gathered by asking them to answer two questionnaires. The first of these had been adapted from a previous study^19^ and was used to obtain sociodemographic, clinical and behavioral information. It contained questions relating to the following variables: age, marital status, place of residence, education level, family income, religion, time of HIV infection, co-infections, time on ART, sexual orientation, condom use, number of sexual partners in the last 12 months, use of licit drugs in the last six months and use of illicit drugs during life. Information on CD4^+^ T-lymphocyte (CD4^+^TL) levels and plasma viral load (VL) were obtained from the participants’ medical records.

The second instrument was the online version in Portuguese of a multidimensional questionnaire assessing adherence to antiretroviral treatment (“Cuestionario para la Evaluación de la Adhesión al Tratamiento Antiretroviral”, iCEAT-VIH).^13^ The main issues addressed in iCEAT-VIH comprised the patient’s compliance with medication intake, antecedents of non-adherence, doctor-patient communication, beliefs about ART, beliefs and expectations about therapeutic efficacy, efforts to follow the treatment, assessment of side effects and level of satisfaction. This questionnaire was completed by the participants in a place in the SCS that had been set aside for this purpose. Both the participant and the researcher were left with copies of the answers placed in the questionnaire.

From the responses given in the online version of this questionnaire (http://www.ceat-vih.info), the ART adherence profile was calculated. At the end of completion of each online questionnaire, a graph with five domains (compliance, history of non-adherence, doctor-patient communication, beliefs and expectations about treatment and satisfaction with treatment) and an overall adherence index, in which scores are transformed onto a scale from 0 to 100, was generated and registered by the participant and, subsequently, by the researcher. To interpret this information, the classification devised by the authors of this questionnaire was used: scores from 0 to 50 = inadequate adherence; scores from 51 to 85 = insufficient adherence; and scores from 86 to 100 = adequate adherence.^13^

### Statistical analysis

All the study data were entered into an Excel database (Microsoft Corporation, Redmond, WA, United States, 2010) and then exported into the SPSS software (IBM, Armonk, NY, United States). Absolute (N) and relative (%) frequencies of the variables were used for descriptions. Odds ratios (OR) and 95% confidence intervals (CI) were used as measurements of the strength of association between low adherence to antiretroviral therapy (outcome), as indicated by inadequate and insufficient levels, and the independent variables. Variables associated with the outcome, with P-value (P) < 0.30 using bivariate analysis, were entered into a backward stepwise logistic regression model (multivariate analysis). P-values < 0.05 were taken to be significant in all analyses. Lastly, statistical analyses were conducted using the SPSS 23.0 software (IBM, Armonk, NY, United States).

### Ethics approval and consent to participate

All participants were included only after providing informed written consent. All procedures were performed in accordance with the relevant guidelines and regulations. This study was approved by the Research Ethics Committee of the Tropical Medicine Center of the Federal University of Pará (Universidade Federal do Pará) in Belém, Brazil (approved on September 13, 2020; CAAE 87450718.0.0000.5172).

## RESULTS

The women’s average age was 37 years. The largest proportions of these subjects were married (47.7%), heterosexual (97.7%), aged between 18 to 35 years (47.7%) and living in the urban area of one of the municipalities in the Rio Caeté integrated region (55.9%); had some religion (94.2%), had low levels of education (illiterate or incomplete elementary school) (63.0%) and were unemployed (69.8%). In addition, many of these women reported that they only had one sexual partner (75.7%), that their partner used a condom during sexual intercourse (46.7%) and that they were not using either licit drugs (68.7%) or illicit drugs (89.6%) (Table 1). More details on the subjects’ socioeconomic, demographic and behavioral characteristics can be seen in the supplementary material (Table S1).

**Table 1.** Sociodemographic, behavioral and therapeutic characteristics of women with human immunodeficiency virus/acquired immunodeficiency syndrome who were treated at the reference unit of the Rio Caeté integrated region (Pará, northern Brazil), in relation to the level of adherence to antiretroviral therapy (ART)

Characteristics	N	ART level	
		Inadequate + Insufficient n (%)	Adequate n (%)
Total	86	69 (80.2)	17 (19.8)
Age (years)			
18-40	55	42 (76.4)	13 (23.6)
More than 40	31	27 (87.1)	4 (12.9)
Marital status			
Not married	45	37 (82.2)	8 (17.8)
Married	41	32 (78.0)	9 (22.0)
Area of residence			
Rural	38	31 (81.6)	7 (18.4)
Urban	48	38 (79.2)	10 (20.8)
Education level			
Up to complete elementary school	61	51 (83.6)	10 (16.4)
High school + university	25	18 (72.0)	7 (28.0)
Work status			
Without job	60	49 (81.7)	11 (18.3)
With job (including retired)	26	20 (76.9)	6 (23.1)
Has a sexual partner			
Yes	71	59 (83.1)	12 (16.9)
No	15	10 (66.7)	5 (33.3)
Number of sexual partners in the last 12 months			
More than one	6	6 (100.0)	0
Up to one	80	63 (78.8)	17 (21.2)
Use of alcohol in the last 12 months			
Yes	23	17 (73.9)	6 (26.1)
No	63	52 (82.5)	11 (17.5)
Use of illicit drugs in life			
Yes	77	61 (79.2)	16 (20.8)
No	9	8 (889)	1 (11.1)
Plasma viral load (copies/ml)			
Detected (≥ 40 copies)	40	37 (92.5)	3 (7.5)
Not detected (< 40 copies)	46	32 (69.6)	14 (30.4)
CD4^+^ T lymphocyte count (cells/mm^3^)			
Up to 350	30	27 (90.0)	3 (10.0)
More than 350	56	42 (75.0)	14 (25.0)
Number of pills a day			
More than one	47	37 (78.7)	10 (21.3)
Only one	39	32 (82.1)	7 (17.9)
Length of time with HIV diagnosis			
Up to 5 years	51	39 (76.5)	12 (23.5)
More than 5 years	35	30 (85.7)	5 (14.3)
Length of time on ART			
Up to 5 years	59	45 (76.3)	14 (23.7)
More than 5 years	27	24 (88.9)	3 (12.5)
Level of satisfaction with ART			
Dissatisfied	15	14 (93.3)	1 (6.7)
Satisfied	71	55 (74.5)	16 (22.5)

A general profile of the responses relating to ART that the participants provided in the iCEAT-VIH questionnaire can be seen in Table 2. Most of the women (79.0%) had insufficient adherence to antiretroviral treatment and obtained scores between 51 and 85 points (average = 75.4) in the iCEAT-VIH. On the other hand, 17 women (19.8%) obtained scores above 85 points (average = 91.0) and were therefore classified as presenting adequate adherence. Only one woman (1.2%) was classified as presenting inadequate adherence, with a score of 28 points.

**Table 2.** Description of data collected regarding adherence to antiretroviral therapy, highlighting the most frequent option among the responses to each of the questions answered by women living with the human immunodeficiency virus/acquired immunodeficiency syndrome (HIV/AIDS) in the Rio Caeté integrated region, Pará, northern Brazil

Questions	Highest frequency response	N (%)
Have you ever stopped taking your medication?*	Not once	57 (66.3)
Have you ever felt better and stopped taking your medication?*	Not once	74 (86.0)
Have you ever felt worse after taking your medication and stopped taking it?*	Not once	73 (84.9)
Have you ever felt sad or depressed and stopped taking your medication?*	Not once	75 (87.2)
How is the relationship you have with your doctor?	Good	72 (83.7)
How much effort do you make to follow (comply) with your treatment?	Much	35 (40.7)
How much information do you have about the medicines you take for HIV/AIDS?	Little	32 (37.2)
How much benefit can the use of these medicines bring you?	Much	45 (52.3)
Do you think your health has improved since you started taking HIV/AIDS medications?	Quite	29 (33.7)
To what extent do you feel able to continue with the treatment?	Much	59 (68.6)
Do you usually take medication on time?	Yes	48 (55.8)
When the test results are good, does your doctor usually use them to give you encouragement and motivation to continue with the treatment?	Yes	74 (86.0)
How do you feel in general about your treatment since you started taking your medication?	Satisfied	47 (54.7)
How do you rate the intensity of side effects relating to the use of HIV/AIDS drugs?	Nothing intense	43 (50.0)
How much time do you think you spend taking your medication?	Short time	46 (53.5)
What assessment do you have of yourself regarding taking HIV/AIDS medications?	Very respectful	44 (51.2)
How much difficulty do you have in taking medication?	Little difficulty	64 (74.4)

*In the last seven days.

In addition, for the majority of the women (53.5%), their VL in their last laboratory test had been undetectable (< 40 copies/ml). The highest VL recorded for any of the participants was 163 copies/ml. Regarding CD4^+^TL levels, the range observed was from 44 to 2,041 cells/mm^3^. The majority of the women (65.1%) had levels of at least 350 cells/mm^3^ (Table 1). The average length of time since receiving the diagnosis of HIV infection was five years, with a range from 1 to 18 years. A majority of the women (45.3%) used only one pill and only 15 participants (17.4%) had had any opportunistic infections in the last 12 months.

The bivariate analysis indicated that only the variable “plasma viral load” was associated with low adherence to ART (Table 3). In addition, a multivariate analysis was performed using age, educational level, having a sexual partner, plasma viral load, CD_4_^+^ T-lymphocyte count, time since diagnosis of HIV infection and length of ART use. Again, only the variable “plasma viral load” was associated with low adherence to ART among these women in the Rio Caeté integrated region (supplementary material; Table S2).

**Table 3.** Results from bivariate analysis on factors relating to low adherence to antiretroviral therapy (inadequate + insufficient) among women living with human immunodeficiency virus/acquired immunodeficiency syndrome (HIV/AIDS) in the Rio Caeté integrated region, Pará, northern Brazil

Factors	Low adherence to antiretroviral therapy	
	OR (95% CI)	P-value
Age (years)		
More than 40	2.1 (0.6-7.1)	0.24
Up to 40	1.0	
Marital status		
Not married	1.3 (0.5-3.8)	0.63
Married	1.0	
Area of residence		
Rural	1.2 (0.4-3.4)	0.78
Urban	1.0	
Education level		
Up to complete elementary school	2.0 (0.7-6.0)	0.22
High school + university	1.0	
Work status		
Without job	1.3 (0.4 - 4.1)	0.61
With job or retired	1.0	
Has a sexual partner		
Yes	2.5 (0.7-8.5)	0.16
No	1.0	
Number of sexual partners in the last 12 months		
More than one	197.39 (0.0 - ∞)	0.88
Up to one	1.0	
Use of alcohol in the last 12 months		
Yes	0.6 (0.2-1.9)	0.38
No	1.0	
Use of illicit drugs in life		
Yes	2.1 (0.2-18.0)	0.50
No	1.0	
Plasma viral load (copies/ml)		
Detected (≥ 40 copies)	5.4 (1.4-20.5)	0.01
Not detected (< 40 copies)	1.0	
CD4^+^ T lymphocyte count (cells/mm^3^)		
Up to 350	3.0 (0.8-11.4)	0.10
More than 350	1.0	
Number of pills a day		
More than one	0.8 (0.3-2.4)	0.70
Only one	1.0	
Length of time with HIV diagnosis		
Up to 5 years	0.6 (0.2-1.7)	0.29
More than 5 years	1.0	
Length of time on ART		
Up to 5 years	0.4 (0.1-1.5)	0.18
More than 5 years	1.0	
Level of satisfaction with ART		
Dissatisfied	230.9 (0.0 - ∞)	0.81
Satisfied	1.0	

OR = odds ratio; CI = confidence interval; ART = antiretroviral therapy

## DISCUSSION

This was one of the first scientific reports on the sociodemographic characteristics and the level of adherence to ART among WLWHA in an area located far from the Brazilian metropolitan regions. It was also the first report from an area in the interior of northern Brazil.

Most of the WLWHA of this study were young (in the reproductive phase) and unmarried, with low levels of education, and had had their diagnoses of HIV/AIDS for approximately five years. These characteristics had already been reported in studies developed in the states of Ceará (northeastern Brazil) and Rio de Janeiro (southeastern Brazil).^20^^,^^21^ According to Silva et al.,^20^ a positive diagnosis of HIV/AIDS can generate a depressed mood and make it difficult to build and maintain affective relationships. This scenario can have a negative impact on women’s lives that may even interfere with their acceptance of the diagnosis and adequate adherence to treatment. This might be even more intense in the context of people with low levels of education and low monthly income, given that these factors could impair their understanding of ART.^14^^,^^15^

Most of the participants in this study lived in the urban areas of municipalities in the Rio Caeté integrated region. This finding (that they mostly lived in urban areas) corroborates what was observed in a study conducted in the municipality of Caxias, state of Maranhão (northeastern Brazil).^1^ Interestingly, a considerable proportion of the women (44.1%) attended at the referral unit lived in the rural areas of these municipalities. This proportion was much higher than the proportion from the rural area that was attended in the municipality of Caxias (10.8%).^1^ This indicates that PLWHA in the interior of Pará are indeed being served by healthcare services. However, the reasons for this difference (in relation to the municipality of Caxias) need to be investigated in the future, in order to provide consistent information for improving healthcare services in the northern and northeastern regions of Brazil, such that more women living in remote areas can be served.

Regarding the sexual behavior of these WLWHA in the interior of Pará, most of them were heterosexual and had only one sexual partner, and their partner used a condom during sexual intercourse. These characteristics are relevant at both the individual and the collective level, since they function as factors for limiting HIV transmission to the sexual partner and, consequently, to the population in general.

At the beginning of the HIV/AIDS pandemic, transmission of the retrovirus was marked by unprotected sex in homosexual relationships and among people with multiple partners.^22^ According to Bertagnoli and Figueiredo,^23^ HIV transmission now occurs more frequently in heterosexual relationships than in homosexual relationships. In the states of Ceará (northeastern Brazil) and São Paulo (southeastern Brazil), WLWHA reported having acquired HIV through heterosexual intercourse with a long-term unprotected partner who they had believed to be faithful, and they used other contraceptive methods.^22^^,^^24^ Another notable behavioral trait among these women in the Rio Caeté integrated region was that they were not using any psychotropic drugs (licit or illicit). Studies have indicated that such behavior is associated with consistent condom use and assists in adherence to ART.^2^^,^^25^^,^^26^

Nonetheless, most of the women in this study were classified as having insufficient adherence to ART. This indicates that there is a need for greater attention from healthcare professionals and institutions that directly care for PLWHA in the interior of Pará. This result is similar to the findings in studies conducted in northeastern, central-western, southeastern and southern Brazil.^14^^,^^27^^,^^28^^,^^29^ In addition, the rate of adequate adherence to ART in this study was lower than what was reported in Equatorial Guinea (42.86%), an African country with a low HDI that is similar to the Rio Caeté integrated region in the Brazilian state of Pará.^29^ Adequate adherence to ART can inhibit HIV replication, thereby resulting in increased CD4+TL counts, which are an important part of the organism’s defenses and also enable improvement of physical resistance to perform work tasks.^5^

Among the variables analyzed in this study, only low plasma viral load was associated with adherence to ART. Thus, care strategies need to be created or improved in order to promote acceptance of and satisfaction with adherence to ART among WLWHA. Concern regarding adherence to ART is a reality in healthcare services and requires multiprofessional intervention. According to Carvalho et al.,^16^ healthcare services need to identify the profile of PLWHA, systematize compliance measures and assess the factors associated with adherence, at regional and even local level, so as to enable early detection of non-adherence to ART and establish the necessary interventions. Low economic status, poverty, illiteracy and low levels of education have been registered as important factors associated with reduced adherence to ART.^1^^,^^15^^,^^29^ These characteristics were observed in the sample of the present study.

Thus, we recommend that the healthcare team’s actions should go beyond care centered on use of drugs and their effects. Healthcare for WLWHA in the interior of Pará needs to include individuals’ characteristics, taking into account their sociodemographic and behavioral profiles in relation to treatments. Through health education, actions that expand people’s knowledge and encourage behaviors that can enhance adherence to ART need to be promoted.

This study had limitations that should be considered. Firstly, the sample size was small and restricted to a single healthcare service, although this data-gathering site is a reference point in assistance for PLWHA in the interior of Pará. In addition, the data-gathering was limited to use of questionnaires. The responses to the questions were statements that were not further investigated, which may have given rise to bias in the information collected. Lastly, scenarios and questions that were misinterpreted by some of the potential participants may have led to their refusal to participate in the study.

## CONCLUSIONS

This study was unique and is very important for the epidemiological scenario of HIV infection in northern Brazil. It identified the sociodemographic, behavioral and clinical characteristics of WLWHA who were seen at a reference unit for HIV/AIDS in the interior of the state of Pará. The characteristics found were similar to those reported in some other Brazilian locations. The participants’ adherence to ART was mostly classified as insufficient. Low plasma viral load was the only variable associated with adherence to ART. It is likely that the participants’ low levels of education and poor socioeconomic conditions interfered with their adherence to ART. This influence may be minimized through multiprofessional interventions that take into account the individuality of the women served by this healthcare service.
